# A rare case of recurrent ovarian cancer presenting as a round ligament metastasis

**DOI:** 10.1186/1477-7819-9-144

**Published:** 2011-11-07

**Authors:** Shinichi Togami, Tomoyasu Kato, Takateru Oi, Mitsuya Ishikawa, Takashi Onda, Shun-ichi Ikeda, Takahiro Kasamatsu

**Affiliations:** 1 Division of Gynecology, National Cancer Center Hospital, 5-1-1 Tsukiji, Chuo-ku, Tokyo 104-0045, Japan

**Keywords:** diagnosis, inguinal mass, ovarian cancer, recurrence, round ligament

## Abstract

We report a rare case of recurrent ovarian cancer presenting as a round ligament metastasis. A 44-year-old woman presented with a lower abdominal mass. Computed tomography showed a pelvic mass. Primary surgery was performed. A histopathological examination showed an ovarian serous adenocarcinoma of Stage IIIb. The patient received 6 cycles of paclitaxel and carboplatin. Almost 2 years after the initial operation, the patient noticed a left inguinal mass. Computed tomography showed a left inguinal mass, 18 mm in size. An excisional biopsy was performed and the tumor was found to originate in the left round ligament. A histopathological examination showed serous adenocarcinoma and there was no evidence of lymph node tissue. Recurrence of ovarian cancer in the round ligament is extremely rare. This unique case suggests, however, that the round ligament in rare cases may be a recurrence site for ovarian cancer, and that accurate differentiation including confirmation by diagnostic imaging and excisional biopsy, is necessary for a definitive pathological diagnosis.

## Background

The majority of women with ovarian cancer present with advanced stage disease. A complete clinical remission after surgical cytoreduction and platinum-based chemotherapy can be achieved in 80-90% of these patients. Despite this, 70-90% of patients will develop recurrent disease [1]. Fifty-five percent of the first relapse cases were found at the pelvis or abdomen [2]. There was a wide variety among the other recurrent sites, such as, retroperitoneal nodes, liver or spleen, brain, and bone [2,3]. We experienced a case with solitary recurrence at the left round ligament. To the best of our knowledge, this is the first report of recurrent ovarian cancer occurring in the round ligament.

## Case

**Figure 1:**
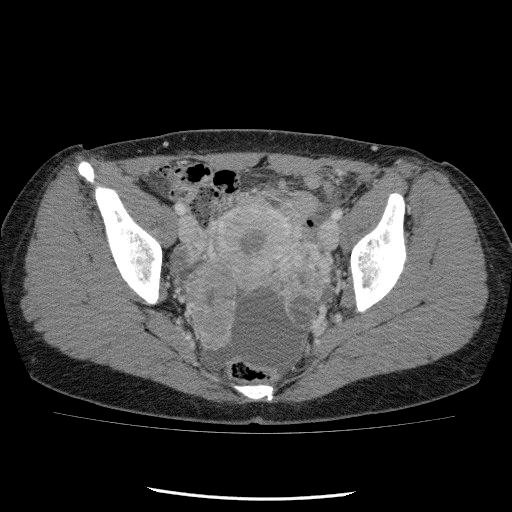
**Enhanced axial CT showing bilateral adnexal tumors**.

A 44-year-old woman presented with a 1.5-year history of progressive enlargement of a lower abdominal mass. On physical examination, a pelvic mass was noted. Transvaginal ultrasound revealed bilateral adnexal masses (left: 80*58 mm, right: 54*40 mm) with solid components in the pelvic cavity. Computed tomography (CT) (Figure 1) and magnetic resonance imaging (MRI) showed bilateral adnexal tumors and dissemination extending from the pelvis to the upper abdomen. There was no evidence of lymphadenopathy. A left mammary tumor was incidentally discovered by CT, and fine-needle aspiration of the breast revealed cells consistent with adenocarcinoma. The serum cancer antigen 125 (CA125) level was elevated to 348 U/ml (normal range, < 35 U/ml). She underwent a total abdominal hysterectomy with bilateral salpingo-oophorectomy, omentectomy, periaortic lymph node biopsy, splenectomy and left partial mastectomy. On intraoperative examination, her surgeons noted involvement of the omentum, spleen and dissemination into Douglas pouch. There was no dissemination involving the diaphragm, liver, paracolic gutters, uterus or peritoneum surrounding the bilateral round ligaments. After primary debulking surgery, she had microscopic residual disease in the Douglas pouch. The pathologic specimen showed extension of the tumor throughout the fallopian tubes, spleen, and omentum. The body of the uterus and the bilateral round ligaments around were not involved in the tumor. The final pathologic diagnosis of the tumor was the International Federation of Gynecology and Obstetrics (FIGO) Stage IIIb ovarian serous adenocarcinoma and left breast invasive ductal carcinoma.

Initially, the patient received 6 cycles of paclitaxel (175 mg/m2) and carboplatin (AUC = 6) for ovarian cancer in April 2009. In August 2009, her serum CA125 levels declined to 5 U/ml. She then received adjuvant radiotherapy (WB 50Gy/25 f) for breast cancer in October 2009. After that, she received routine follow-up from her gynecologic oncologist.

**Figure 2:**
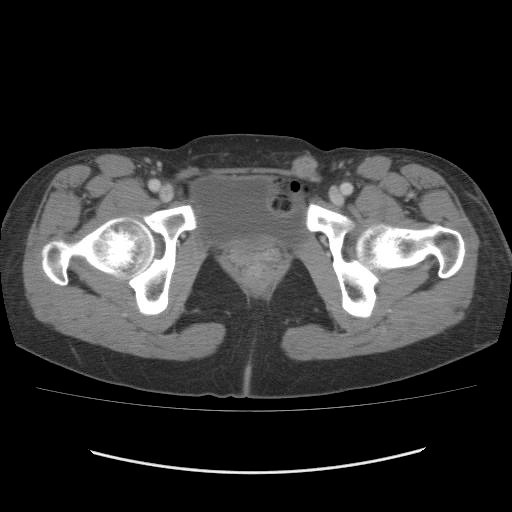
**Enhanced axial CT shows a left inguinal mass, 18 mm in size (arrow)**.

**Figure 3:**
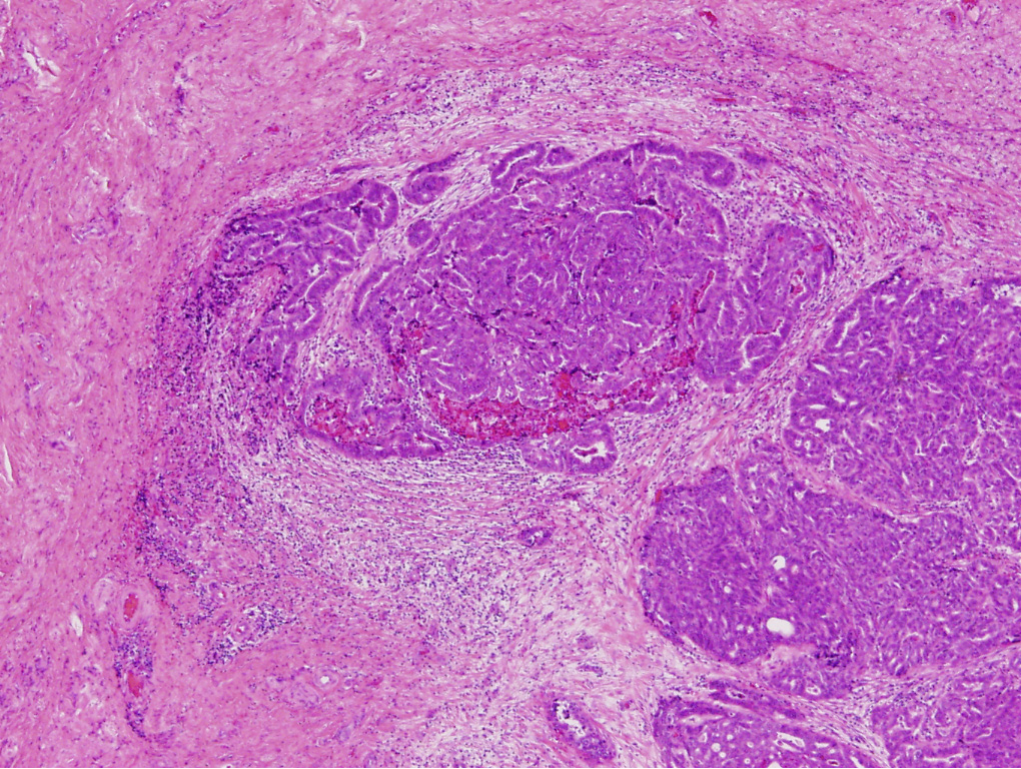
**The histopathological appearance of the round ligament recurrence: Serous adenocarcinoma exists in the context of fibrous connective tissue, including small blood vessels**. (H&E, ×40).

Almost 2 years after her initial ovarian cancer operation, the patient noticed a left inguinal mass. CT showed a left inguinal mass, 18 mm in size (Figure 2), but no abnormal mass in chest, abdomen or pelvis. This mass was located on the fascia and medially to the femoral vein. The serum CA125 level was within normal limits. A fine-needle aspiration of the left inguinal mass revealed cells consistent with adenocarcinoma, but it was difficult to confirm the location of the primary lesion. She underwent an excision biopsy of the left inguinal mass. An incision was made directly over the mass which was present in the left round ligament. At the proximal site of the tumor, a normally appearing left round ligament was exposed. Then the mass was removed entirely. The pathological diagnosis of the tumor was serous adenocarcinoma, which was similar to the prior ovarian cancer, and there was no evidence of lymph node tissue (Figure 3).

## Discussion

The majority of patients with ovarian cancer responds well to the initial treatment, but most of them will develop recurrent disease [4]. Recurrent disease involves most frequently the pelvis or the abdomen [2,3]. This case demonstrates a most unusual recurrence for ovarian cancer, presenting as a round ligament metastasis. The round ligament extends from the uterus, through the inguinal canal, and ends in the region of the mons pubis and labia majora. Embryologically, this is the female equivalent of gubernaculums testis and is predominantly composed of smooth muscle fibers, connective tissue, blood vessels, and nerves with a mesothelial coating [5].

Because round ligament recurrence of ovarian cancer is very rare, we performed a MEDLINE search of the English language literature, but no example could be found. Some unusual tumors involving the round ligament have been reported in the literature: dermoid cyst, endometriosis, mesothelial cyst, leiomyoma and leiomyosarcoma [6-8]. These tumors of the round ligament are very rare developmental disorders, which have been reported as case reports. Especially, leiomyoma is the most common tumor associated with the round ligament [9]. Patil et al. [9] reported the clinicopathological features of 55 cases of women with inguinal smooth muscle tumors of women. Histologically, 23 tumors were considered as leiomyomas, and five tumors arose in the round ligament. In contrast to the leiomyomas, none of the leiomyosarcomas were associated with the round ligament. Indeed, leiomyosarcomas of the round ligament of the uterus are extremely rare and there is only 1 case report published of leiomyosarcoma arising in the round ligament of the uterus [6]. Recurrent tumors of the round ligament are also rare. We found 1 case report of recurrent endometrial cancer stage Ia, originating in the round ligament [10]. The 5 cm solid mass was located near the right superficial inguinal ring. Resection of the round ligament mass and dissection of the retroperitoneal node were performed, and one positive obturator node was found. They concluded that patients presenting with a round ligament recurrence should have a thorough work-up with pelvic lymph node evaluation. In our case, CT showed no evidence of pelvic lymph node swelling, so we performed only the round ligament mass resection to confirm the pathological diagnosis. Postoperative PET/CT scan revealed no hot spot, showing that metastasis to the round ligament was solitary.

Two possible pathways of metastasis to the round ligament have been considered: The lymphatic and the vascular pathway. Pathologically, the tumor showed vascular infiltration but no evidence of lymph node tissue, but it is difficult to determine whether the metastasis is pathway whether lymphatic or vascular. Ovarian cancer generally metastasizes via the lymphatic system or by peritoneal dissemination [11]. Lymphatic vessels enter and travel along the round ligament to reach the inguinal region, and the most likely hypothesis is that the microscopic tumor metastasized to the round ligament through a lymphatic pathway.

There are some reports of solitary splenic metastasis of ovarian cancer after surgical remission [11-13]. In terms of treatment, splenectomy was performed in all cases and adjuvant chemotherapy was administered in most cases. The decision as to whether adjuvant chemotherapy is indicated must be carefully considered in each case.

## Conclusions

This case presents an unusual example of a recurrence site for ovarian cancer. Although solitary ovarian cancer recurrence at the round ligament is extremely rare, it should be included in the differential diagnosis for any patient with a past history of ovarian cancer. The round ligament has the potential to be a site of occurrence of various tumors. Among them, benign tumors such as leiomyoma are often seen in the round ligament. This unique case suggests that the round ligament in rare cases may be a site of recurrence in ovarian cancer, and indicates that accurate differentiation, including confirmation with diagnostic imaging and excisional biopsy, are necessary because the subsequent treatment depends significantly on the pathological results.

## Consent statement

Informed consent was obtained from the patient for publication of this case report and accompanying images. A copy of the written consent is available for review by the Editor-in-Chief of this journal.

## Competing interests

The authors declare that they have no competing interests.

## Authors' contributions

ST, TK and TOi have operated this case. MI, TO, SI and TK have assisted to analyze all data. All authors read and approved the final manuscript.
